# Plant Translation Factors and Virus Resistance 

**DOI:** 10.3390/v7072778

**Published:** 2015-06-24

**Authors:** Hélène Sanfaçon

**Affiliations:** Pacific Agri-Food Research Centre, Agriculture and Agri-Food Canada, 4200 Highway 97, Summerland, BC V0H 1Z0, Canada; E-Mail: helene.sanfacon@agr.gc.ca; Tel.: +1-250-494-6393; Fax: +1-250-494-0755.

**Keywords:** eukaryotic translation initiation factors, recessive resistance, translation repression, plant-virus interactions

## Abstract

Plant viruses recruit cellular translation factors not only to translate their viral RNAs but also to regulate their replication and potentiate their local and systemic movement. Because of the virus dependence on cellular translation factors, it is perhaps not surprising that many natural plant recessive resistance genes have been mapped to mutations of translation initiation factors eIF4E and eIF4G or their isoforms, eIFiso4E and eIFiso4G. The partial functional redundancy of these isoforms allows specific mutation or knock-down of one isoform to provide virus resistance without hindering the general health of the plant. New possible targets for antiviral strategies have also been identified following the characterization of other plant translation factors (eIF4A-like helicases, eIF3, eEF1A and eEF1B) that specifically interact with viral RNAs and proteins and regulate various aspects of the infection cycle. Emerging evidence that translation repression operates as an alternative antiviral RNA silencing mechanism is also discussed. Understanding the mechanisms that control the development of natural viral resistance and the emergence of virulent isolates in response to these plant defense responses will provide the basis for the selection of new sources of resistance and for the intelligent design of engineered resistance that is broad-spectrum and durable.

## 1. Introduction

Although plant viruses encode a number of essential proteins (e.g., coat proteins, movement proteins, replication enzymes) their coding capacity is limited and they must rely on host factors for every stage of the infection cycle [1,2,3]. An early and critical step of this cycle is the translation of viral RNAs. Viruses do not normally encode canonical translation factors, but have developed a wide array of strategies to highjack translation factors from their hosts and favor the translation of viral RNAs to the detriment of endogenous mRNAs [4,5]. Usurped translation factors have also been shown to play critical roles in the replication of viral genomes and even in assisting viral movement in the plant. Perhaps not surprisingly, many naturally occurring plant recessive resistance genes have been mapped to mutations in isoforms of translation initiation factors eIF4E and eIF4G. These mutations generally hinder the interactions of host factors with viral RNAs or proteins [6,7,8,9]. Non-host resistance or resistance to non-adapted virus strains has also been linked with incompatibility between viruses and cellular translation factors [10,11]. New sources of resistance to a number of plant viruses have been developed using a more directed approach aimed at mutating or down-regulating translation factors [1,6]. Finally, translation repression has recently emerged as an endogenous antiviral RNA silencing mechanism [12], reinforcing the idea that hindering the translation of viruses or their ability to interact with translation factors is a promising avenue for virus control. In this review, I will discuss recent advances in our understanding of the interactions between plant viruses and translation factors, the utility of various natural or engineered resistance mechanisms based on the manipulation of plant translation factors, the emergence of virulent virus isolates in response to these new selection pressures and possible future strategies to engineer durable virus resistance.

## 2. Biological Functions of Translation Factors in Plants 

### 2.1. Translation of Cellular mRNAs

**Figure 1:**
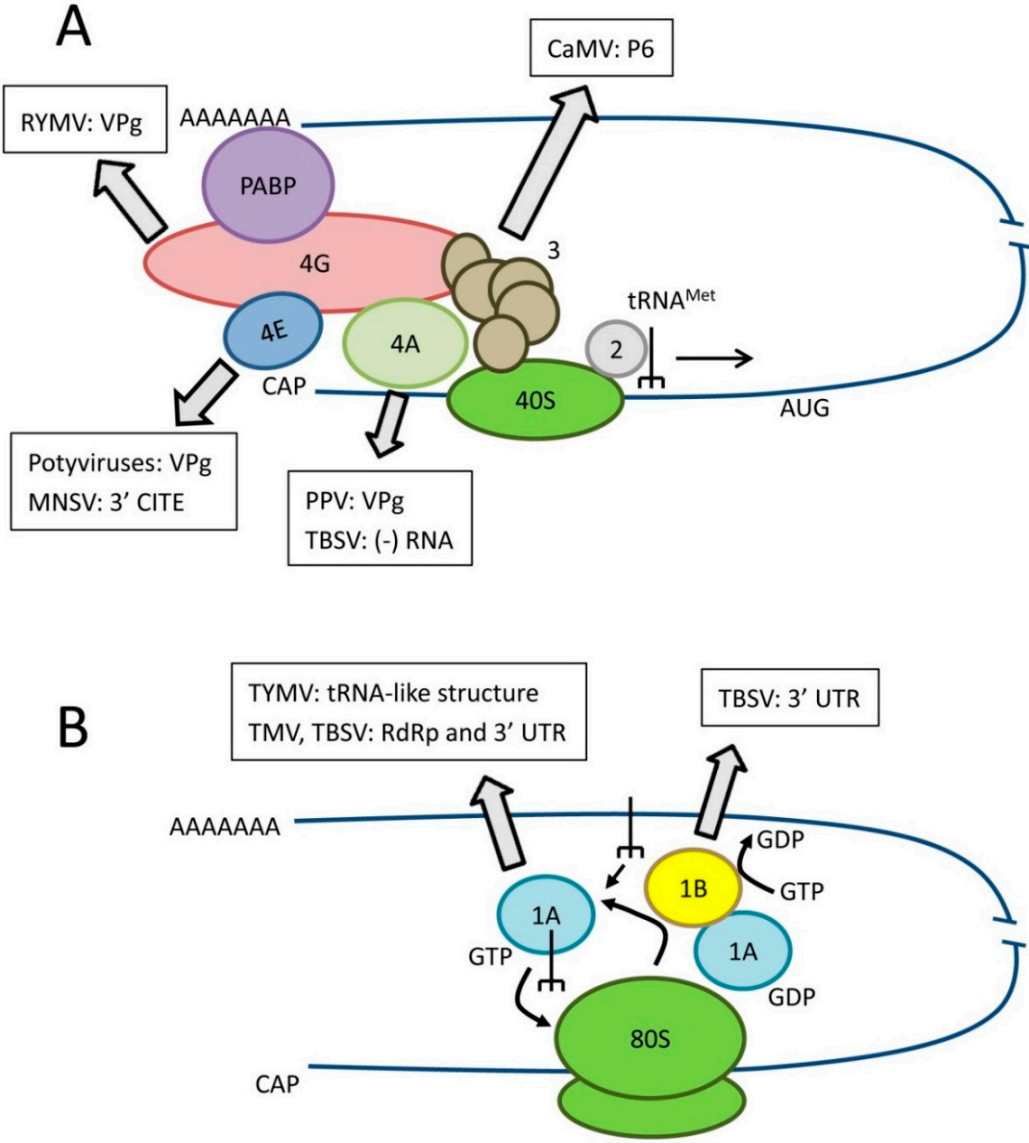
Role of translation factors in canonical eukaryotic translation and interactions with viral RNAs or proteins. (**A**) Simplified diagram of translation initiation. Key initiation factors are shown as well as the best characterized interactions with viral components. eIF3 is shown as a complex of subunits. Please refer to the text for details of the specific isoform of eIF4E or eIF4G or specific eIF4A-like helicase recruited by various plant viruses; (**B**) Simplified diagram of translation elongation. Key elongation factors are shown as well as the best characterized interactions with viral components.

Eukaryotic mRNA translation has been reviewed by others [13,14] and will not be described in detail here. Rather, this section is focused on the biological function(s) of key translation factors that have also been implicated in virus resistance. Translation initiation is a crucial step of protein synthesis that requires a large number of eukaryotic initiation factors (eIFs, Figure 1A). Translation of eukaryotic mRNAs depends on the binding of translation eukaryotic initiation factor 4E (eIF4E) to their 5′ m^7^G cap structure and is also enhanced by interaction of the polyA-binding protein (PABP) with their 3′ polyA tail. The large eIF4G scaffold protein binds to both eIF4E and PABP promoting circularization of the mRNA. The tight association between eIF4E and eIF4G constitute the core of the eIF4F complex. eIF4G also interacts with eIF4A, a DEAD-box ATPase and ATP-dependent RNA helicase. DEAD-box helicases are so-named because they contain a conserved Asp-Glu-Ala-Asp (DEAD) motif. eIF4A unwinds the mRNA to assist ribosome scanning. RNAs with long structured 5′ untranslated regions (UTR) are particularly dependent on eIF4A or other DEAD-box helicases. The eIF4G-eIF4A interaction is looser than that between eIF4G and eIF4E and although eIF4A is considered a component of eIF4F, it is easily lost during purification. Other DEAD-box RNA helicases such as Dhx29 and Ded1/Ddx3 also participate in mRNA translation and may have distinct specificities for mRNAs with specific features in their 5′ UTR. eIF4E, eIF4G, eIF4A and other DEAD-box RNA helicases are key translation factors frequently usurped/manipulated by viruses and are attractive targets for antiviral strategies (Section 3, Figure 1A). The eIF3 protein complex (11–12 subunits, depending on the organism) interacts with both eIF4G and the 40S ribosome subunit, thereby bringing the 43S pre-initiation complex (which includes the 40S subunit and the eIF2-GTP-tRNA^Met^ ternary complex) to the mRNA. As discussed below, a subunit of eIF3 has been implicated in the translation reinitiation mechanism of cauliflower mosaic virus (CaMV) polycistronic mRNA.

In plants, two isoforms of eIF4F are present: eIF4F, which consists of eIF4E and eIF4G, and eIFiso4F, which includes eIFiso4E and eIFiso4G [15,16,17,18]. To add to the complexity, these isoforms are often encoded by multi-gene families. For example, there are 1–4 genes coding for eIFiso4G depending on the plant species (two for *Arabidopsis thaliana*) [17]. eIF4E and eIFiso4E, although of similar size, only share 50% amino acid sequence identity. eIF4G (~180 kDa) and eIFiso4G (~86 kDa), share common binding motifs for eIF4E, eIF4A and eIF3 in their C-terminal domains but differ in size due to a large truncation of the N-terminal region in eIFiso4G. In wild-type *A. thaliana* cells, only cognate complexes (*i.e*. eIF4E-eIF4G and eIFiso4E-eIFiso4G) accumulate in detectable amounts [19] and in wheat germ extracts, eIFiso4F is 3–5 time more abundant than eIF4F [20]. Knock-out of eIFiso4G in *A. thaliana* causes severe defects in plant health. In contrast, knock-out or down-regulation of eIF4E or eIFiso4E is tolerated and plants show little signs of impairment [21,22]. Interestingly, knock-out of eIFiso4E is compensated by increased expression of eIF4E, suggesting functional redundancy between the two isoforms [21]. Indeed, mixed complexes can be formed *in vitro*, although with lower binding activity than that shown for the cognate complexes, and these hetero-complexes are active in translation [23]. In spite of some functional redundancy, eIF4F and eIFiso4F exhibit distinct specificity for mRNAs *in vitro* [24]. As will be described below, viruses have adapted to use either eIF4F or eIFiso4F and mutation of one isoform of eIF4E or eIF4G can lead to resistance to plant viruses without affecting the plant health, making them attractive sources of natural or engineered resistance. Similarly to mammalian cells, plant translation initiation complexes utilize not only eIF4A (eIF4A-1 or eIF4A-2, which share 96% amino acid sequence identity) but also a variety of other DEAD-box RNA helicases. In proliferating *A. thaliana* cells, eIF4A1/2 is the predominant helicase found in association with both eIF4F and eIFiso4F complexes [19]. In quiescent cells, other DEAD-box helicases also co-purify with these complexes. The partial functional redundancy of eIF4A-like helicases in plants also makes them amenable to manipulation towards virus resistance.

Once translation is initiated, eukaryotic elongation factors (eEFs) allow continued synthesis of the nascent polypeptide chain [25] (Figure 1B). Eukaryotic elongation factor 1A (eEF1A also referred to as eEF1α in the literature) is an abundant cytoplasmic protein that forms a ternary complex with GTP and aminoacylated tRNAs (all tRNAs but the initiator tRNA^Met^) and delivers these tRNAs to the elongating ribosome. As discussed below, eEF1A is also frequently found in association with the replication complexes of positive-strand RNA viruses (Section 3.2). eEF1A functions with the eEF1B complex, a guanine nucleotide exchange factor, which helps recycling eEF1A into its active GTP-bound form.

### 2.2. Additional Biological Functions of Translation Factors 

In addition to their roles in the canonical translation of cellular mRNAs, translation factors have other biological functions that are of interest for viruses. In mammalian cells, a sub-population of eIF4E accumulates in nuclear bodies and has been implicated in the regulated nuclear export of mRNAs that contain a specific structure (the 4E-sensitivity element) [6,26,27]. In *A. thaliana* cultured cells, eIF4E is predominantly cytoplasmic although its partition to the nucleus is regulated by the cell growth cycle [19]. eIFiso4E is equally distributed in nucleus and cytoplasm. Thus, a role for the plant eIF4E and/or eIFiso4E in nuclear export is likely. The plant eIFiso4F complex also associates with microtubules and facilitates their end-to-end annealing, suggesting a role in the regulation of microtubule dynamics [28,29]. The microtubule localization of eIFiso4F depends on a direct interaction between eIFiso4G and microtubules [28]. eEF1A, another multitask protein, may regulate nuclear export, cytoskeleton and microtubule stabilization/organization and protein stability, likely via interactions with the proteasome [30]. Viruses exploit these activities to control the stability of their proteins, regulate their replication and facilitate their movement in the cell and from cell-to-cell.

## 3. Characterization of the Interactions between Cellular Translation Factors and Plant Viruses and Identification of Potential Sources of Viral Resistance 

### 3.1. Translation Factors Facilitating Non-Canonical Viral RNA Translation

As mentioned above, viruses depend on cellular translation factors to synthesize their proteins. The RNAs of positive-strand RNA viruses differ from plant mRNAs in that they lack the 5′ cap structure and/or the polyA tail. Viral RNAs often have highly structured 5′ UTRs with multiple stem-loops or pseudoknots that are necessary for other steps of the infection cycle (replication, encapsidation) and that can interfere with normal scanning of ribosomes. Finally, some viruses have polycistronic RNAs that require translation reinitiation mechanisms or internal ribosome entry sites to facilitate translation at downstream open reading frames (ORFs). Thus, viruses have developed a variety of non-canonical mechanisms to translate their RNAs. The reader is referred to a collection of excellent recent reviews that describe these mechanisms in detail [4,5,31,32,33,34,35,36]. 

#### 3.1.1. Core eIF4F/iso4F Components Recruited by Viral RNAs or Viral VPg Proteins

One common strategy used for the translation of non-canonical viral RNAs is the recruitment of initiation factors (usually eIF4E/iso4E or eIF4G/iso4G) or ribosomal RNAs by *cis*-acting translation enhancing sequences (CITE) present on the viral RNAs. These *cis*-acting sequences include internal ribosome entry sites (IRES) in the 5′ untranslated region (UTR) or in intergenic regions, and other CITE sequences in the 3′ UTR or even within an ORF. Long-distance kissing loop interactions between *cis*-acting sequences in the 5′ and 3′ UTRs are often required for translation enhancement by promoting circularization of the RNA. A natural melon recessive resistance gene to melon necrotic spot virus (MNSV, a carmovirus) corresponds to a mutation of eIF4E that disrupts its interaction with 3′ CITE sequences [37] and will be described in detail below (Section 4.2). In addition, non-host resistance to MNSV in *Nicotiana benthamiana* was recently reported to be caused by an incompatibility between the viral RNA and eIF4E [11]. 

The uncapped RNAs of some viruses, notably members of the families *Potyviridae* and *Secoviridae* and of the genus *Sobemovirus* are covalently linked with a small protein (termed VPg) at their 5′ end [38]. Although variable in size and sequence, the VPg of these divergent viruses share the common property of being intrinsically disordered, possibly allowing flexible adaptation to various binding partners [38,39,40,41,42]. An interaction between the turnip mosaic virus (TuMV, a potyvirus) VPg protein and eIFiso4E, first identified in a yeast two-hybrid screen [43], was later shown to be essential for virus infection [44]. As will be discussed in detail below, recessive resistance to potyviruses has been mapped to mutations of eIF4E and/or eIFiso4E (Section 4.1, Table 1). In addition, resistance to one strain of plum pox virus (PPV, another potyvirus) in wild-type *A. thaliana* and *Chenopodium foetidum* was attributed to the inability of this isolate to recruit translation initiation factors [10]. Potyvirus RNA translation depends on an IRES located in the 5′ UTR, which recruits one of the eIF4F isoforms, eIF4F in the case of tobacco etch virus (TEV) [45]. The VPg protein promotes host translational shut-down by competing with eIF4E or eIFiso4E cap binding activity and enhances the affinity of eIF4E/iso4E for viral RNAs *in vitro*, thereby facilitating their translation [44,46,47]. Ectopic expression of VPg was shown to inhibit the translation of cellular mRNAs and stimulate the translation of potato virus A RNA *in planta* [48]. In addition to VPg and eIFiso4E, the ribosomal stalk protein P0 was also implicated in viral RNA translation, and silencing of P0 reduces the translation of a reporter viral RNA [49]. In addition, analysis of *A. thaliana* T-DNA insertion lines revealed the dependence of potyviruses on specific eIF4G isoforms that work concomitantly with the corresponding eIF4E isoform [50], suggesting recruitment of the entire eIF4F complex, possibly for translation of viral RNAs. Interestingly however, resistance to TEV in *A. thaliana* eIFiso4E deficient plants is not correlated with defects in viral RNA translation [51]. Thus, although the VPg-eIF4E/iso4E interaction may support viral RNA translation in some potyvirus-host combinations, it is likely that it also facilitates other steps of the infection cycle, notably viral RNA replication and/or cell-to-cell movement [6,38]. This is discussed below (Section 3.2 and Section 3.3). The VPg protein of rice yellow mottle virus (RYMV, a sobemovirus) was found to bind directly with eIFiso4G rather than eIF4E isoforms [52]. As will be discussed below (Section 4.2), natural recessive resistance to RYMV in rice is correlated with mutation of eIFiso4G. It is not known whether the VPg-eIFiso4G interaction plays a role in the translation of the viral RNA or in other aspects of the viral infection cycle. The role played by the VPg protein of secovirids and its possible interactions with translation factors is less clear. The VPg protein of secovirids (2–4 kDa) is smaller than that of potyviruses or sobemoviruses (10–26 kDa). The VPg-protease polyprotein of tomato ringspot virus (a nepovirus), was shown to bind eIFiso4E *in vitro*, and although the eIFiso4E-binding domain was within the protease region, binding to eIFiso4E was enhanced by the presence of the VPg [53]. However, the biological function of the interaction is not known. 

#### 3.1.2. eIF4A-Like DEAD-Box Helicases Assisting the Translation of Bromovirus and Tombusvirus RNAs

Using a yeast replication model, DED1, a DEAD-box helicase related to eIF4A and implicated in yeast mRNA translation, was shown to facilitate the translation of the RNA2-encoded viral RNA-dependent RNA polymerase (RdRp) from brome mosaic virus (BMV, a bromovirus) [54]. DED1 interacts with a unique *cis*-acting sequence present only in the 5′ UTR of RNA2. Thus the translation of RNA1 and RNA3 was not affected by DED1. Of interest, mutation of DED1 (*ded1-18* mutant with two amino acid substitutions) hinders the accumulation of the 2a RdRp and reduces virus replication but does not affect yeast mRNAs translation or yeast growth. Although this is an attractive candidate for bromovirus resistance, it is not known whether mutation of an equivalent helicase in plant would have a similar effect on BMV accumulation. Knock-down of the yeast DED1 also impacted the translation of tomato bushy stunt virus (TBSV, a tombusvirus) RNA [55]. Interestingly and as described below, DED1 and other DEAD-box RNA helicases also play important roles in the regulation of viral RNA replication in yeast and in plants (Section 3.2). 

#### 3.1.3. Recruitment of eEF1A by tRNA-Like Structure at the 3′ End of Viral RNAs

The RNAs of several plant viruses (e.g., tobamoviruses, tymoviruses and bromoviruses) have a tRNA-like structure at their 3′ end rather than a polyA tail [56]. These structures are recognized by aminoacyl-tRNA synthetases and become aminoacylated. The aminoacylated tRNA-like structure of turnip yellow mosaic virus (TYMV, a tymovirus) interacts with eEF1A and with ribosomes [57,58] and has been shown to enhance translation of the viral RNA [59]. As will be discussed below, the interaction of eEF1A with the TYMV tRNA-like structure also regulates its replication 

#### 3.1.4. Recruitment of an eIF3 Subunit and Associated Factors by Cauliflower Mosaic Virus for Translation Re-Initiation

A distinctive feature of some viral RNAs is that they are polycistronic or contain small ORFs in their 5′ UTRs. Leaky scanning, ribosome shunting or reinitiation mechanisms allow tight regulation of the translation rate of downstream ORFs [60]. The CaMV transactivator p6 protein facilitates translation re-initiation downstream of the 35S RNA large and highly structured 5′ UTR that also contains multiple small ORFs [61]. P6 interacts with a subunit of eIF3, various ribosomal proteins and a new plant translation factor, RISP, which itself interacts with the 60S ribosome [60,62,63,64]. The CaMV p6 protein also binds and activates the serine/threonine protein kinase TOR (target-of-rapamycin), which phosphorylates and activates RISP [65]. These interactions were shown to facilitate the translation re-initiation mechanism *in vitro* and *in vivo*. An *A. thaliana* T-DNA insertion line that prevent the expression of *RISPa*, the gene encoding RISP, shows only modest resistance to CaMV, probably due to the presence of a second highly homologous gene (*RISPb*) with overlapping function [63]. Unfortunately, double knock-out of *RISPa* and *RISPb* is not viable. The *A. thaliana* 35-7 line, which is silenced for TOR expression by RNA interference (RNAi), seemed more promising as it displayed strong resistance to CaMV [65]. Interestingly, two *A. thaliana* lines silenced for TOR expression (including the 35-7 line) are partially resistant to watermelon mosaic virus (a potyvirus) but fully susceptible to TuMV [66]. Treatment of wild-type plants with AZD-8055, an ATP-competitive inhibitor of TOR, also resulted in partial resistance to watermelon mosaic virus but not to TuMV [66]. Thus, manipulation of TOR expression or activity may provide a useful source of resistance not only to CaMV (and possibly other caulimoviruses) but also to some potyviruses. Whether such resistance is transferable to crops of economic importance and whether it can be stable in a field situation will require further study. 

### 3.2. Translation factors Regulating Viral RNA Replication

In addition to their obvious role in viral RNA translation, cellular translation factors are recruited to membrane-associated viral replication complexes (VRCs) and regulate viral RNA replication. In many viruses, translation and replication are tightly linked and viral RNAs or proteins are brought to the VRCs co-translationally, along with translation factors [67,68,69]. Some translation factors interact with viral RdRps and are recruited to VRCs; e.g., eIF3 subunits for BMV and tobacco mosaic virus (TMV) [70,71] and eEF1A for TMV and TuMV [72,73]. Recruitment of translation factors to VRCs can also require interactions with *cis*-acting sequences on viral RNAs (see below) or with viral proteins other than the RdRp, for example the potyvirus VPg protein. The TuMV VPg, or rather the VPg domain in the larger membrane-targeted 6K-VPg-protease polyprotein, was initially shown to recruit eIFiso4E to the VRC suggesting an as yet-undefined role for this factor in viral RNA replication [74]. Later, other translation factors were also found in association with TuMV VRCs, including PABP, a DEAD-box helicase and eEF1A [72,75,76]. DEAD-box helicases and eEF1A are discussed in detail below. The biological function of PABP association with potyvirus VRCs is not known, although it may enhance replication by allowing circularization of the viral RNA (jointly with eIF4E and eIF4G isoforms). *A. thaliana* T-DNA insertion lines that knock-out the expression of PABP show increased resistance to TuMV without visibly affecting plant growth or health [77], providing a possible new source of resistance to potyviruses.

#### 3.2.1 Recruitment of DEAD-Box RNA Helicases to Tombusvirus and Potyvirus VRCs

DEAD-box RNA helicases, related to eIF4A, are recruited to tombusvirus VRCs [55]. Contrary to larger RNA viruses, tombusviruses do not encode a helicase to facilitate their replication. In yeast, DED1 binds to the 3′ end of the TBSV (−) strand RNA, is recruited to the VRCs and enhances (+) strand RNA synthesis by facilitating the unwinding of the (−) strand RNA [55]. The selective binding of DED1 to (−) strand RNA allows assymetric RNA synthesis and accumulation of excess (+) strand RNA for new rounds of translation/replication or for encapsidation. Overlapping functions were assigned to the yeast DBP2 and *A. thaliana* RH20 DEAD-box helicases that, similarly to DED1, bind to the 3′ end of TBSV (−) strand RNA to stimulate (+) strand RNA synthesis [78]. A second group of DEAD-box helicases including the yeast eIF4AIII-like Fal1p and DDX5-like Dbp3p helicases and the orthologous plant AtRH2 and AtRH5 helicases (from *A. thaliana*) also participate in the selective enhancement of TBSV (+) strand RNA synthesis by binding to another *cis*-acting replication enhancing element in the 5′ terminal region of the (−) strand RNA [79]. This allows unwinding of this highly structured region of the RNA. Overexpression of AtRH2 and AtRH5 in *N. benthamiana* results in enhanced viral accumulation and symptom severity [79]. Although down-regulation or mutation of these plant helicases may provide resistance to tombusviruses, this has not been tested. 

Using a yeast-two hybrid screen, the VPg protein from PPV was shown to interact with a peach DEAD-box helicase (PpDXL) closely related to *A. thaliana* eIF4A genes [75]. Homozygous *A. thaliana* lines with T-DNA insertion in eIF4A genes could not be generated, likely because of pleiotropic effects of these mutations. However, knock-out of another related DEAD-box helicases, AtRH8, resulted in increased resistance to two potyviruses (PPV and TuMV) without visibly impacting the plant [75]. Interaction between AtRH8 and VPg was confirmed and AtRH8 was found in association with VRCs in TuMV-infected cells. The exact function of the cellular helicase in potyvirus VRCs is not known, as contrary to tombusviruses, potyviruses do encode a helicase, the CI protein, which is also recruited to the VRC [75]. Of practical interest, ectopic overexpression of a truncated AtRH8 protein, corresponding to the VPg-binding domain, also suppresses virus accumulation, probably by outcompeting the wild-type protein for VPg interaction [75]. This dominant-negative approach may be applicable to other helicases that interact with tombusviruses or other viruses.

#### 3.2.2. Regulation of Tymovirus, Tobamovirus and Tombusvirus Replication by eEF1A 

Potyviruses, tobamoviruses and tombusviruses recruit eEF1A to their VRCs [72,80,81]. I have already discussed the role of eEF1A in promoting the translation of the TYMV RNA by binding to the 3′ terminal tRNA-like structure. Interestingly, the TYMV tRNA-like structure also acts as a promoter for (−) strand RNA synthesis. eEF1A was shown to down-regulate (−) strand RNA synthesis, probably by competing with the RdRp for binding to the tRNA-like structure [82]. It was suggested that conformation changes in the TYMV tRNA-like structure may regulate binding to eEF1A or RdRp at different stages of the infection cycle.

The multiple functions of eEF1A in tombusvirus RNA replication have been studied in details although primarily in the yeast model system [81,83]. eEF1A interacts with the TBSV RdRp and also binds to a *cis*-acting element in the 3′ UTR of the RNA. These interactions are thought to stimulate the recruitment of viral RNAs to the replication complex. eEF1A also helps stabilizing the p33 accessory protein and enhances (−) strand RNA synthesis. Screening of eEF1A mutants in yeast identified several mutants that could not stimulate viral replication, thereby resulting in partial resistance to TBSV [81,83]. However, the utility of these mutants in establishing virus resistance is tempered by their pleiotropic effects on host translation accuracy or efficiency. Perhaps of greater practical interest, another elongation factor eEF1Bγ, a member of the eEF1B complex and a non-essential cellular protein, is also associated with the TBSV VRCs [84]. eEF1Bγ was shown to interact with a loop in a tight stem-loop structure of the TBSV RNA 3′ UTR. The binding helps loosening the structure and stimulates (−) strand RNA synthesis synergistically with eEF1A. Silencing of eEF1Bγ expression in *N. benthamiana* plants resulted in severe reduction of TBSV RNA accumulation and symptom amelioration. The silenced plants also displayed partial resistance to TMV, suggesting a potential for the development of broad-spectrum resistance [84]. 

In the case of tobamoviruses such as TMV, eEF1A also interacts with both the RdRp and with a *cis*-acting element in the viral RNA (a pseudoknot in the upstream region of the 3′ UTR) [73,85]. In *N. benthamiana*, eEF1A is encoded by at least six genes corresponding to two groups of related sequences [86]. Using the 3′ UTR sequence of representative eEF1A genes to specifically silence genes belonging to only one of the two groups, partial resistance to TMV was achieved with only moderate impairment of plant growth [86]. Silencing of all forms of eEF1A simultaneously, which improves virus resistance, also results in severe plant growth defects. The resistance is apparently due to reduced viral replication and/or cell-to-cell movement. It was suggested that the known interaction of eEF1A with the cytoskeleton may contribute to the assembly of VRCs or to the intracellular and/or cell-to-cell movement of these complexes. In a separate study, a virus-induced gene silencing approach using a tobacco rattle virus vector was used to down-regulate the expression of eEF1A and eEF1B(α/β) [87]. Partial resistance to TMV was achieved as evidenced by reduced virus accumulation. eEF1A and eEF1B were both shown to interact with the RdRp. Although it is likely that eEF1B accumulates in the VRCs, its exact biological function in viral replication or another step of the infection cycle is not known. 

### 3.3. Other Roles for Translation Factors in Enhancing the Virus Infection Cycle

Most interactions described above regulate the translation or replication of viral RNAs. However, in some cases, and as already discussed for the TMV-eEF1A interaction, other steps of the infection cycle are apparently regulated by translation factors. Plants deficient for eIF4E show restricted cell-to-cell movement of some potyviruses, possibly related to the microtubule-binding affinity of the eIF4G binding partner of eIF4E [88,89]. In addition to binding the VPg, eIFiso4E was also found to bind the TEV coat protein and facilitate systemic movement of the virus in the plant [51]. Finally, eIF4E/iso4E is also found in the nucleus/nucleolus in infected cells, together with the nuclear VPg-protease polyprotein [74]. Possible roles for the nuclear localization of eIF4E/iso4E in mRNA transport, host gene regulation and RNA silencing have been suggested [6]. 

### 3.4. Translation Repression as an Emerging Antiviral RNA Silencing Mechanism in Plant

RNA silencing is a ubiquitous gene regulation mechanism and a well-documented antiviral defense in plants [12,90,91]. Small RNAs incorporated into RNA-induced silencing complexes (RISCs) provide the sequence specificity of RNA silencing. Argonaute enzymes (AGO), the main component of RISCs, can catalyze the degradation of target RNAs or they can repress their translation. While translation repression of plant mRNAs directed by microRNAs (miRNAs) or small-interfering RNAs (siRNAs) is now well-documented [92,93,94,95,96,97], it is only recently that translation repression was proposed as an alternative RNA silencing mechanism operating against plant viruses [98,99,100,101]. Translation repression of viral mRNAs was first observed in association with the defense response activated following the interaction between a dominant resistance gene and a viral elicitor and was shown to be dependent on AGO4 [99]. Another example of AGO-dependent translation repression mechanism came from the analysis of *N. benthamiana* plants infected with tomato ringspot virus [100]. In this interaction, symptom recovery follows an initial symptomatic systemic infection. The translation of one of the viral RNAs was found to be repressed in late phases of infection. Down-regulation of AGO1 relieves the translation repression and prevents symptom recovery.

Translation repression has been best studied in fly and mammalian cells and is mediated by imperfect base-pairing of miRNAs to target mRNAs [102]. In animal cells, miRNAs normally bind to the 3′ UTRs of target mRNAs and direct not only translation repression but also mRNA destabilization, which is initiated by deadenylation/decapping enzymes. An AGO binding partner, the GW182 protein, participates in both processes using a number of distinct pathways. Importantly, translation repression can occur independently of mRNA destabilization and does not strictly require GW182 [103]. Under conditions in which deadenylation is prevented, translation repression was found to target very early steps of translation initiation, e.g., the assembly of the eIF4F complex [104]. 

The mechanism of translation repression in plant cells is less well understood. Translation repression of cellular mRNAs by miRNAs or siRNAs depends on AGO1 or AGO10 and usually requires perfect or near-perfect base pair complementarity [93]. There are no homologs of GW182 in plants, although a distinct protein (SUO) also with GW motifs, is required for miRNA-directed translation repression [96]. A detailed *in vitro* study revealed several distinct translation repression mechanisms in plants [94]. Consistent with the absence of GW182 in plants, deadenylation of mRNAs was not observed in tobacco cell lysates. Binding of miRNAs is not only restricted to the 3′ UTR of the mRNAs but also to the 5′ UTR and even the ORF. When bound to the 3′ UTR, the translation repression mechanism functions in a manner similar to that observed in animal cells likely by preventing eIF4F assembly/function. Interestingly, binding of miRNAs to targets within the ORF functioned by a different mechanism by preventing translation elongation [94]. The identity of small RNAs (if any) controlling AGO-dependent translation repression of viral RNAs is not known. In addition, the potential role played by specific initiation or elongation factors during antiviral translation repression remains to be characterized.

## 4. Practical Applications of Antiviral Resistance Based on Mutations or Down-Regulation of Translation Factors

As discussed above, translation factors influence multiple steps of the virus infection cycle, as exemplified for eIF4E, eIF4A-like helicases and eEF1A. This presents welcome opportunities to develop multifaceted antiviral strategies. However, in many cases mutations of translation factors also have unwanted consequences to the plant physiology. In fact, only in a few instances have manipulation of translation factors resulted in demonstrated applications for virus resistance in the field. In the next two sections, I will discuss well-documented examples of field resistance based on mutations of eIF4E or eIF4G isoforms. 

### 4.1. Endogenous and Engineered Resistance to Potyviruses Based on Defective eIF4E or eIFiso4E 

The family *Potyviridae* is one of the largest families of plant viruses and includes many economically important pathogens of cultivated crops, especially members of the genus *Potyvirus* [105]. Cultivated crops and wild plant species have co-evolved with potyviruses, resulting in the development of recessive resistance. In most cases characterized so far, natural recessive resistance to potyviruses has been mapped to mutations of eIF4E or eIFiso4E genes. The long co-existence of plants and potyviruses has also given rise to the emergence of virulent isolates that have adapted to circumvent the recessive resistance [106,107]. An extensive body of literature documents the characterization of natural recessive resistance genes and the intelligent design of new resistance strategies based on disrupting the interaction between potyviruses and eIF4E/iso4E [108]. This information is summarized in Table 1 and only key points will be discussed here. 

Natural recessive resistance to potyviruses is generally associated with mutations of eIF4E or eIFiso4E that hinder their interaction with the VPg protein. Most of these mutations have been mapped to two surface-exposed regions of eIF4E near the cap-binding pocket [106,107]. However, mutations in the pepper eIF4E *pvr1* allele that conferred resistance to TEV were not strictly correlated with reduced cap-binding activity [109]. *Trans*-complementation studies of naturally occurring mutated eIF4E alleles (pea *sbm1* and lettuce *mo1* resistance genes) with overexpressed wild-type or mutated forms of eIF4E have also shown that mutants of eIF4E isoforms with reduced interactions with potyviruses are not necessarily hindered in their ability to bind to the cap structure or to other translation factors [106,110,111]. Thus, at least in some cases, mutated eIF4E isoforms implicated in virus resistance are fully functional in cellular mRNA translation. In addition to the characterized recessive resistance genes, other sources of resistance can be found in cultivated crops or wild plant species by screening for natural or induced mutated alleles of eIF4E isoforms using techniques such as Targeting-Induced Local Lesions In Genome (tilling), eco-tilling (screening of targeted natural variation) and next generation sequencing [112,113,114,115,116]. Silencing of an eIF4E isoform by transgenic expression of small intron-spliced hairpins with homology to the endogenous gene has been successfully used as an alternative approach to engineer resistance to one or several potyviruses [117,118,119]. This technology has been applied not only to model herbaceous hosts but also to fruit trees (e.g., resistance to PPV in plum) [119]. Finally, overexpression of mutated eIF4E (or eIFiso4E) alleles has been validated as an efficient technology to transfer resistance from one plant species to another [120,121,122,123]. In at least one case, the overexpressed recessive allele conferred dominant resistance [120]. 

The potyvirus VPg protein interacts specifically with either eIF4E or eIFiso4E and this specificity is influenced by the specific host-virus combination and can even vary from one virus strain to another [50,124,125,126]. As a result, mutation of a single eIF4E isoform is often sufficient to provide resistance to a target potyvirus. Using yeast two-hybrid screens, *in vivo* bimolecular fluorescence complementation assays or *in vitro* co-immunoprecipitation methods to identify the specific plant eIF4E isoform-VPg interaction for the virus under study can be a useful preliminary step before engineering potyvirus resistance based on manipulation of eIF4E isoforms. However, some potyviruses have been shown to recruit either eIF4E or eIFiso4E and down-regulation or mutation of both isoforms may be necessary for durable resistance [115,127,128,129]. In addition and as discussed in Section 2.1, many plants have multiple functional copies of eIF4E (or eIFiso4E) genes that may be used specifically or interchangeably by potyviruses. For example, some *Brassica rapa* lines have three copies of eIFiso4E and TuMV was shown to use at least two of these genes [129]. Similarly, distinct potyviruses interact with tomato eIF4E1 and/or eIF4E2 genes and simultaneous silencing of both genes was required to provide broad-spectrum resistance to potyviruses [118]. 

Virulent potyvirus isolates have been described that overcome recessive resistance genes corresponding to eIF4E/iso4E alleles. In most cases, virulence has been mapped to mutations in the VPg protein [130,131,132]. Although in some cases, mutations in the VPg increase its binding affinity to the resistant eIF4E/iso4E protein [106], in other cases it does not [88,109,133]. In at least one case, mutation of VPg does not increase its affinity for other eIF4E isoforms either, eliminating the possibility that other isoforms of eIF4E are used by the virulent virus [133]. Rather, it was suggested that the mutated VPg may bind to other viral or host factors, associated with the VPg-eIF4E complex. In agreement with this suggestion, the lettuce *mo1* resistance gene was shown to be overcome by mutations in the CI protein in virulent lettuce mosaic virus (LMV) isolates [134,135]. The CI protein is a multifunctional protein that interacts with both VPg and eIF4E and mutations that confer virulence also strengthen complex formation [136]. The CI-VPg-eIF4E complex may be necessary for viral replication and/or movement, possibly in association with eIF4G and microtubules [6,137]. Finally, the clover yellow vein virus (ClYVV) P1 protein has also been implicated in the overcoming of the pea *cyv2* resistance gene, although the mechanism is not known [138]. Taken together, these results indicate that the interaction of potyviruses with eIF4E isoforms is complex and can be overcome by mutations in various regions of the viral genome. 

Can this information be used towards the generation of durable resistance? Analysis of the co-evolution between potato virus Y (PVY) and eIF4E recessive resistance alleles in *Capsicum* species did not reveal clear trends, possibly because of pleiotropic effects of mutations in the VPg protein, making the prediction of virulence emergence difficult [107,139]. However, it was suggested that eIF4E mutations that provide broad-spectrum resistance to PVY isolates may be better candidates for durable resistance. Recently, a series of eIFiso4E variants were designed based on amino acids implicated in other potyvirus-eIF4E/iso4E interactions [123]. Overexpression of these variants in transgenic *Brassica rapa* was shown to provide broad-spectrum resistance to TuMV isolates that may also be more durable.

**Table 1:** Resistance to potyvirids based on eIF4E or eIFiso4E.

Transl. Factor	Plant Species	Cause of Resistance	Virus ^1^	References
eIF4E	*Capsicum spp*	Natural resistance genes *pvr1*/*pvr1^2^* (point mutations)	PVY, TEV, PepMoV	[89,106,109,112,115,130,131,132,139,140,141]
eIF4E	*Lactuca sativa*	Natural resistance gene *mo1* (point mutations)	LMV	[111,142]
eIF4E	*Pisum sativum*	Natural resistance genes *sbm1*/*wlv*/*cyv2* (point mutations)	PsBMV, BYMV, ClYVV	[88,110,114,143,144]
eIF4E	*Phaseolus vulgaris*	Natural resistance gene *bc-3* (point mutations)	BCMV, ClYVV	[145,146]
eIF4E	*Solanum habrochaites*	Natural resistance gene *pot-1* (point mutations)	PVY, TEV	[147]
eIF4E	*Hordeum vulgare*	Natural resistance genes *rym4/rym5* (point mutations)	BaMMV, BaYMV	[148,149,150,151,152,153]
eIF4E	*Citrullus lanatus*	SNP mapping with resistance (point mutations)	ZYMV	[154]
eIF4E	*Prunus armeniaca*	One of several QTLs associated with resistance	PPV	[155]
eIF4E	*Arabidopsis thaliana*	EMS mutagenesis (*cum1-1*: inserted stop codon)	ClYVV	[126]
eIF4E	*Solanum lycopersicum*	EMS mutagenesis and TILLING (*SleIF4E1-G1485A*: splicing mutant)	PVY, PepMoV	[116]
eIF4E	*Cucumis melo*	RNAi	MWMV, ZYMV, CVYV (+ a carmovirus)	[117]
eIF4E	*Solanum lycopersicum*	RNAi targeting eIF4E-1 and eIF4E-2	PYV, TEV, PepMoV + 4 more potyviruses	[118]
eIF4E	*Solanum lycopersicum*	Overexpression of *pvr1* allele from *Capsicum* (dominant resistance)	TEV, PepMoV	[120]
eIF4E	*Solanum tuberosum*	Overexpression of *pvr1^2^* allele from *Capsicum*	PVY	[121]
eIF4E	*Solanum tuberosum*	Overexpression of mutated *S. tuberosum* eIF4E	PVY	[121]
eIF4E	*Solanum tuberosum*	Overexpression of wild potato eIF4E-1 allele *Eva1* and RNAi of endogenous eIF4E-1	PVY	[122]
eIFiso4E	*Capsicum spp*	Natural resistance gene *pvr6* (functions with *pvr1 or pvr1^2^)* (deletion causing truncation of protein)	PVMV, ChiVMV	[115,128]
eIFiso4E	*Brassica rapa*	Natural resistance gene *retr01* (splicing mutant)	PPV, TuMV	[156,157,158]
eIFiso4E	*Arabidopsis thaliana*	EMS mutagenesis (*lsp1-1*, *lsp1-2*, *lsp1-3*: inserted stop codon)	TuMV	[159]
eIFiso4E	*Arabidopsis thaliana*	Transposon (Spm) mutagenesis (*AteIFiso4E-1*: insertion in first exon)	TuMV, LMV	[21,129]
eIFiso4E	*Prunus domestica*	RNAi	PPV	[119]
eIFiso4E	*Brassica rapa*	Overexpression of mutated proteins	TuMV	[123]

^1^ Virus abbreviations not defined in the text: PepMoV (pepper mottle virus), PsBMV (pea seed-borne mosaic virus), BYMV (bean yellow mosaic virus), BCMV (bean common mosaic virus), BaYMV (barley yellow mosaic virus), BaMMV (barley mild mosaic virus), ZYMV (zucchini yellow mosaic virus), MWMV (Maroccan watermelon mosaic virus), CVYV (cucumber vein yellowing virus), PVMV (pepper vein mottling virus), and ChiVMV (Chilli veinal mottle virus). All viruses listed belong to the genus *Potyvirus* with the exception of BaMMV and BaYMV which are members of the genus *Bymovirus*.

### 4.2. Resistance to Other Plant Viruses Conferred by Mutations of eIF4E or eIF4G Isoforms

Natural or induced resistance conferred by mutations/knock-out of eIF4F isoforms (eIF4E or eIF4G) is not restricted to members of the family *Potyviridae* but can also target a variety of (+) strand RNA viruses including carmoviruses, cucumoviruses, sobemoviruses and waikaviruses. This information is summarized in Table 2. 

The natural resistance to a sobemovirus conferred by a mutation in eIFiso4G is an interesting case. Sobemoviruses although unrelated to potyvirids also have a VPg protein linked to the 5′ end of their RNAs (see Section 3.1.1). Resistance to RYMV in rice was correlated with mutations in eIFiso4G, corresponding to several alleles of *rymv1* naturally found in *Oryza sativa* (*rymv1-2*) or *O. glaberrima* (*rymv1-3*, *rymv1-4*, *rymv1-5*) [160,161]. The two rice species are cultivated in different regions of Africa and are infected by separate groups of RYMV isolates. Screening of a large collection of RYMV isolates and directed evolution studies using serial passaging of susceptible isolates identified mutations in the VPg as the main source of resistance breaking against *rymv1-2* and *rymv1-3* [52,162,163,164,165]. All characterized mutations are located in the central domain of the VPg. However, mutations that allow breaking of *rymv1-2* or *rymv1-3* are distinct and very few isolates are able to overcome both alleles [162]. In fact, although virulent isolates emerge rapidly and have a selective advantage in resistant rice cultivars, they are often less well-adapted to susceptible varieties [166]. It was suggested that adaptation to one resistant allele may prevent further mutations to adapt to a second divergent allele, limiting the emergence of virulent isolates in the field [162]. In sobemoviruses, the VPg is initially expressed as a polyprotein p2a that also includes other protein domains. Recently, characterization of a RYMV *rymv1-*2-breaking isolate identified mutations in a region of p2a that is distinct from the VPg domain, although the biological function of this protein domain is not known [167].

Another well-characterized natural recessive resistance is that of the melon *nsv1* eIF4E allele against melon necrotic spot virus (MNSV, genus *Carmovirus*, family *Tombusviridae*) [168]. The MNSV RNA genome is neither capped, nor polyadenylated and is also not bound to a VPg. Virulence in the MNSV-264 strain is due to mutations in the 3′ UTR, more specifically in a CITE sequence [37,169]. The 3′ CITE from the avirulent MNSV strain can only promote translation in resistant melon cells when the eIF4E allele from susceptible melon is provided *in trans* [37]. In contrast, the 3′ CITE from the MNSV-264 strain directs effective translation in both susceptible and resistant melon. Interestingly, the mutated 3′ CITE of MNSV-264 also allows translation and virus replication in *N. benthamiana*, a plant normally not susceptible to MNSV. Thus, *N. benthamiana* non-host resistance to avirulent MNSV was correlated with an incompatibility between the 3′ CITE and NbeIF4E [11]. Of particular interest, the recent characterization of a new resistance breaking MNSV isolate (MNSV-N) revealed acquisition of the 3′ CITE from a member of the family *Luteoviridae* [170]. Artificial creation of chimeric viruses had already demonstrated that exchange of unrelated 3′ CITE sequences is possible and can result in productive infection [171]. In the case of MNSV-N, the acquired 3′ CITE functions by a completely distinct mechanism and does not require interaction with eIF4E as demonstrated by its ability to efficiently promote translation in melons that are silenced for eIF4E [170]. Interfamily recombination is likely a rare event but, at least in this particular case, led to resistance breaking of recessive resistance.

**Table 2:** Resistance to non-potyvirids based on isoforms of eIF4E or eIF4G.

Transl. Factor	Plant Species	Cause of Resistance	Virus ^1^	References
eIFiso4G	*ryza spp.*	Natural resistance gene *rymv1* (several alleles with distinct point mutations)	RYMV (sobemovirus)	[52,160,161,162,163,164,165,166,167,172,173]
eIF4G	*Oryza sativa*	Natural resistance gene *tsv1* (point mutations)	RTSV (waikavirus)	[174]
eIF4G	*A. thaliana*	EMS-induced mutation *cum2*	CMV (cucumovirus) + TCV (carmovirus)	[175]
eIF4E	*Cucumis melo*	Natural resistance gene *nsv1* (point mutations)	MNSV (carmovirus)	[37,168,169,170]
eIF4E	*Cucumis melo*	RNAi	MNSV + 3 potyviruses	[117]
eIF4E	*Cucumis spp.*	Natural resistance identified by Eco-TILLING	MNSV	[113]
eIF4E	*A. thaliana*	EMS-induced knock-out *cum1*	CMV (cucumovirus)	[175]

^1^ Virus abbreviations not defined in the text: RTSV (rice tungro spherical virus), CMV (cucumber mosaic virus), TCV (turnip crinkle virus)

## 5. Concluding Remarks 

Characterization of natural recessive virus resistance has provided new insights on the interactions between plant viruses and translation factors and on the selection pressures controlling the emergence of virulent isolates. In addition, the systematic characterization of interactions between viral RNAs or proteins and translation factors has identified new potential targets for antiviral resistance. As discussed in Section 4.1, it is hoped that this knowledge can be used in the future to identify new sources of resistance in wild plant species that could be transferred to cultivated crops. In addition, specific mutations likely to provide broad-spectrum resistance can be selected and engineered in characterized translation factors. In that respect, the new CRISPR-Cas9 genome editing technology should allow targeted introduction of specific mutations in eIF4E isoform genes in plants for which natural resistance is not available [176]. It will be interesting to follow the evolving interactions between plant viruses and translation factors as new resistant varieties are implemented in the field.
